# Difficult airway management and the novice physician

**DOI:** 10.4103/0974-2700.58668

**Published:** 2010

**Authors:** Noble L Aikins, Rajpaul Ganesh, Kurt E Springmann, Jeffrey J Lunn, Joanne Solis-Keus

**Affiliations:** Texas Tech University Health Science Center (TTUHSC) El Paso, TX, USA

**Keywords:** Airway, difficult, physician

## Abstract

**Background:**

Selection of the ideal airway device in patients with difficult airways (DA) or potentially difficult airways remains controversial, especially, for a novice anesthesia physician (NP) who must deviate from conventional direct laryngoscopy with a rigid laryngoscope following a failed intubation and employ one of the several alternative devices. The author determines and compares tracheal intubation success rates, times to success and complications of a novice physician using four alternative airway devices in 20 obese (BMI more than 27.5) patients who may be more difficult to intubate than normal weight patients.

**Materials and Methods:**

In this prospective randomized experimental study the author investigates a novice physician's use of the Bullard™, Fiberoptic™, Fastrach™ and Trachlight™ comparing reliability, rapidity and safety of orotracheal intubations. Following induction of anesthesia the NP was allowed up to a maximum of two attempts per device at oral intubation. Mean intubation times plus/minus SD, per cent success rates and postoperative complications were evaluated for each device.

**Results:**

The Fastrach™ was successful 100% of the time on the first attempt requiring a mean time of 55 seconds plus/minus 6.6. All intubations were unsuccessful following two attempts with the Fiberoptic™. A success rate of 20% (one of five) was achieved with the Trachlight™ on first attempt after 95 seconds. The Bullard™ was successful in 40 % (two of five) of the patients after a mean time 60 seconds plus/minus five, but was the only device to result in mild oral discomfort one day post operatively.

**Conclusions:**

In the hands of a novice physician managing a difficult or potentially difficult airway, often encountered in obese patients, the Fastrach™ demonstrated the highest success rate.

## INTRODUCTION

Management of a difficult airway (a “difficult airway” is defined as the clinical situation where a trained anesthesiologist experiences problems with mask ventilation, tracheal intubation or both)[1] is challenging to any anesthesiologist but can be especially daunting to a novice anesthesia physician (NP). Following failed oral intubation attempts with a rigid laryngoscope (Macintosh or Miller blade), the NP (one with five or less {based on empirical evidence} practical experiences with an airway device on human subjects) must often use an alternative airway device. Four recognized devices include the Bullard laryngoscope (Bullard™ ACMI Corporation, Southborough, MA), Fiberoptic bronchoscope (Fiberoptic™, Olympus Corporation; Tokyo, Japan), intubating laryngeal mask (Fastrach™; LMA North America. Inc., San Diego, CA) and Lightwand device (Trachlight™, Laerdal Medical, Armonk, NY). The supervising anesthesiology faculty often determines the alternative airway device based on personal preference and experience. There is limited objective data guiding an experienced anesthesiologist in selection of the most efficient device for the NP. Our goal is to examine intubations with these four alternative airway devices on difficult or potentially difficult airways in order to mimic a typical scenario encountered in clinical practice; therefore, our study consist of obese (BMI greater than 27.5) patients which may be more difficult to intubate compared to normal-weight patients.[2–4]

## MATERIALS AND METHODS

After IRB approval, 20 obese patients, ASA II-III, were included in this study. The primary investigator is a board-certified anesthesiology faculty possessing experience with all four airway devices in 20 or more patients. The laryngoscopist studied is a resident anesthesia physician with more than 10 months experience using a conventional laryngoscope. The resident physician viewed instructional videotape and received didactic instruction with the four devices before trials and did not have more than five practical experiences with the four airway devices. Patients whose BMI is more than 27.5, without contraindications to mask ventilation, (i.e. gastrointestinal disease, recent trauma) were selected by the primary investigator. Written informed consent was obtained from all subjects before beginning the study.

The trial was not performed in cases of difficulty with mask ventilation following induction of anesthesia. The patients included in this study were randomly assigned the Bullard™ group, Fiberoptic™ group, Trachlight™ group or Fastrach™ group. Following 30cc oral Bicitra (0.2mg glycopyrrolate intravenously, Fiberoptic™ group) and preoxygenation for three to five minutes, general anesthesia was induced intravenously with one to two mg/kg propofol, one to three mcg/kg fentanyl and one mg/kg of succinylcholine IV. After induction, cricoid pressure was maintained and mask ventilation verified. In all groups, an appropriate sized endotracheal tube (ett) for oral intubation was chosen (inner diameter, 7.5-8.0mm in males and 7.0-7.5mm in females).

In the three groups, Bullard™, Trachlight™ and the Fiberoptic,™ two attempts limited to a maximum time of 120 seconds were allowed. If these two attempts did not result in a successful intubation it was judged a failure.

In the Fastrach™ group a size number 4 or 5 laryngeal mask was chosen depending on the patient's size. Two attempts to insert laryngeal mask portion were allowed. Following insertion the cuff was inflated with 25-40 ml of air and manual ventilation attempted. Tidal volumes more than 10ml/kg, adequate movement of the chest wall and over 15 cm H 20 airway pressure were judged acceptable ventilation. Only one attempt at blind intubation through the Fastrach™ with the silicone ett was undertaken. If unsuccessful, one attempt at fiberoptic bronchoscope guided tracheal intubation through the Fastrach™ was allowed. Inability to insert the Fastrach™ and intubate the tracheal in 120 seconds was designated a failure.

For all groups, the number of attempts and time elapsed before tracheal intubation (time of insertion of test device into oropharnyx to manual ventilation through the tracheal tube) was recorded. The BMI, age and intubation time are reported as mean plus/minus SD. Complications such as mucosal bleeding injury, hoarseness, dental injury, sore throat, difficult or painful swallowing were assessed by patient review postoperatively and on postoperative day one. All continuous data of the groups were analyzed using one-way ANOVA. Nominal data was analyzed using *X*2 contingency table. *P* < 0.05 is considered statistically significant.[5]

## RESULTS

Twenty patients were enrolled for investigation in this study. No patients refused to participate. No patients were withdrawn because of difficult mask ventilation or other mechanical complications. All patients had Mallampati scores of two or three. No significant differences occurred in the BMI or the age between the groups [Table 1]. All patients classified as failure to intubate were successfully intubated with a rigid laryngoscope by the NP on the first attempt in less than thirty seconds. Each group consists of five patients, for a total of 20 patients studied.

**Table 1:** Comparative results of 4 groups

Device	Fastrach™	Bullard™	Trachlight™	Fiberoptic™
BMI	34 ±7	35 ±4.5	31 ±1.6	32 ±2.5
Intubation time (sec.)	55 ± 6.6	60 ±5	95	-
Success rate (%)	100	40	20	0
Safety (1-5*)	5*	4*	5*	5*
Age (years)	46.8 ±9	40 ±17	43 ±14	48.4 ±16

SUCCESS RATE SIGNIFICANTLY HIGHER WITH FASTRACH (P<0.05); SAFETY: HIGHEST=5*; LOWEST=1*

A significantly higher overall success rate was demonstrated with the Fastrach (*P* < 0.05). All (100%) of the patients in the Fastrach™ group were intubated on the first attempt (insertion of the mask and blind intubation with the endotracheal tube) in a mean intubation time of 55 seconds plus/minus 6.6 [Table 1].One minor complication included a small airflow obstruction in one patient following insertion of the Fastrach mask which was resolved in less than five seconds by applying the “Aikins Maneuver”.

The Bullard™ group showed an overall success rate of 40% (40% on first attempt and 0% on second attempt). The mean intubation time was 60 seconds plus/minus five. This group recorded the only postoperative complication for the study. One of the five patients complained of mild oral discomfort on postoperative day one, which resolved the next day (Table 1—highest level of safety indicated by 5* and lowest 1*).

In the Trachlight™ group, the overall success rate was 20% (first attempt 20% and 0% second attempt). The intubation time was 95 seconds [Table 1].

All the subjects in the Fiberoptic™ group could not be intubated after two attempts [Table 1].

## DISCUSSION

Past studies have examined the novice physician's use of a single alternative airway device in normal-weight patients or compared trained physician's experience with one or two alternative airway devices in patients with normal anatomy or cervical spine disorders.[6–11] To this author's knowledge, this is the first study to examine a NP's use of four well-known alternative airway devices to intubate obese patients.

Obese patients may be more difficult to intubate than normal-weight patients.[2–4] Other factors have been associated with difficult laryngoscopy such as sternomental distance, thyromental distance, large neck circumference, limited neck extension/flexion, decreased mouth opening, receding mandible and prominent teeth.[12,13] However, no single factor has been shown to conclusively effect successful intubations.[14,15] Obesity (as measured by BMI), was the selection criteria used to enroll patients in the study because it is easily measured and, other than facial/neck abnormalities, obesity may represent the single most common condition leading anesthesiologists to have a heightened concern for potential or actual airway difficulty. The airway devices in our study may be useful for performing neutral orotracheal intubation in patients with unstable cervical spines.[6,16,17] However, the main focus here was to provide an objective basis for identifying the best device for the inexperienced physician encountering a difficult airway or failed intubation with a rigid laryngoscope.

This study demonstrates that, in the hands of the novice physician, the Fastrach™ may be the most reliable alternate airway device for orotracheal intubation of obese patients. Successful intubations were significantly higher with the Fastrach than the other devices. The lowest mean intubation time was recorded with the Fastrach™. The Fastrach™ mask was inserted successfully on the first attempt and all intubations were performed blind on first attempt. Based on the findings, this device seems valuable not only as an elective but also as an emergency airway device following failed intubation with other techniques. Our data showed impressive results. However, one of the patients in the Fastrach™ group experienced a mild obstruction following Fastrach™ mask placement which was alleviated by the “Aikins Maneuver”. This maneuver involves a gentle upward force at the angle of the mandibles bilaterally after Fastrach™ mask insertion if resistance to ventilation is encountered. This maneuver may allow the Fastrach™ to acquire a more optimal position without manipulating or reinserting the mask itself. This technique should not be used for patients with unstable cervical spines.

The Fiberoptic™ may reduce cervical spine movement,[17] but the device demonstrated no usefulness with time-regulated oral intubation by the novice physician. The novice physician was not successful following two attempts in any of the subjects. Perphaps a nasal approach would have increased the success rate. This approach requires additional time and preparation and, for the purposes of our study, does not represent a clinically feasible technique to rapidly secure a difficult airway. Cricoid pressure used in our study protocol may have also affected the success rate of the Fiberoptic™ compared with other devices.[9] The novice physician often reported large amounts of mucosal tissue causing poor views and impeding acquisition of the glottic opening. This finding was not unexpected, as novice physicians frequently encounter difficulty maneuvering the insertion tube of the fiberoptic through the oral pharynx and into the glottic opening.

The Bullard™ was the second most rapid and reliable device (60seconds plus/minus five and 40% success on the first attempt and zero per cent success on the second attempt) and the least safe (brief, mild oral discomfort postoperative day one). As noted by the investigator, the view with the Bullard™ was often distorted by redundant mucosal tissue and lens fogging. Difficulty was also encountered with manipulation of the stylet while guiding the endotracheal tube. These results differ from some other reports comparing the Bullard™ to the Fastrach™[10]; however, in this study, intubations with the Fastrach™ were faster because all intubations were conducted blind without fiberoptic guidance.

The Trachlight™ took more time to intubate (95 seconds first attempt) and was less reliable (20% success rate) than the Fastrach™ and the Bullard™. Although the Trachlight handle and battery were replaced before trials, the light appeared dim and dull once placed in the patient's oral pharynx and did not improve with manipulation in the areas of the piriform fossa and hypopharynx. Our results differed from another report comparing the Fastrach™ to the Trachlight™ by well-trained (10-years in practice) personnel with no Trachlight™ or Fastrach™ experience in patients with unstable cervical spines.[6] Perhaps the amount of redundant tissue in the obese patients we studied affected the brightness of the Trachlight™ dimishing the effectiveness of the device compared to normal weight patients used in other studies.

## CONCLUSIONS

The Fastrach™ demonstrated the greatest success rate of the alternative airway devices used by the NP. Our findings provide trained anesthesia staff objective data when deciding which airway device is most suited for the novice physician, especially, if the intubation is more urgent than elective. Nevertheless, this study is greatly limited by its size (number of NP and number of subjects). For instance, individual novice physicians in a residency training program may demonstrate varying levels of proficiency with different devices. Therefore, this study serves as an impetus and a model for future large scale studies.
